# YangXue QingNao Wan and Silibinin Capsules, the Two Chinese Medicines, Attenuate Cognitive Impairment in Aged LDLR (+/-) Golden Syrian Hamsters Involving Protection of Blood Brain Barrier

**DOI:** 10.3389/fphys.2018.00658

**Published:** 2018-06-01

**Authors:** You-Yu Gu, Ping Huang, Quan Li, Yu-Ying Liu, George Liu, Yu-Hui Wang, Ming Yi, Li Yan, Xiao-Hong Wei, Lei Yang, Bai-He Hu, Xin-Rong Zhao, Xin Chang, Kai Sun, Chun-Shui Pan, Yuan-Chen Cui, Qing-Fang Chen, Chuan-She Wang, Jing-Yu Fan, Zhi-Zhong Ma, Jing-Yan Han

**Affiliations:** ^1^Department of Integration of Chinese and Western Medicine, School of Basic Medical Sciences, Peking University, Beijing, China; ^2^Tasly Microcirculation Research Center, Peking University Health Science Center, Beijing, China; ^3^Key Laboratory of Microcirculation, State Administration of Traditional Chinese Medicine of the People’s Republic of China, Beijing, China; ^4^Key Laboratory of Stasis and Phlegm, State Administration of Traditional Chinese Medicine of the People’s Republic of China, Beijing, China; ^5^State Key Laboratory of Core Technology in Innovative Chinese Medicine, Beijing, China; ^6^Key Laboratory of Molecular Cardiovascular Sciences, Institute of Cardiovascular Sciences, Peking University Health Science Center, Beijing, China; ^7^Neuroscience Research Institute, Peking University, Beijing, China; ^8^Department of Anatomy, Peking University Health Science Center, Beijing, China

**Keywords:** familial hypercholesterolemia, low density lipoprotein receptor, blood–brain-barrier, cognitive, YangXue QingNao Wan, Silibinin capsules

## Abstract

The purpose of the study was to explore the effect and the underlying mechanism of YangXue QingNao Wan (YXQNW) and Silibinin Capsules (SC), the two Chinese medicines, on cognitive impairment in older people with familial hyperlipidaemia. Fourteen month-old female LDLR (+/-) golden Syrian hamsters were used with their wild type as control. YXQNW (0.5 g/kg/day), SC (0.1 g/kg/day), or YXQNW (0.5 g/kg/day) + SC (0.1 g/kg/day) were administrated orally for 30 days. To assess the effects of the two drugs on plasma lipid content and cognitive ability, plasma TC, TG, LDL-C, and HDL-C were measured, and Y maze task was carried out both before and after administration. After administering of the drugs for 30 days, to evaluate the effect of the two drugs on disturbed blood flow caused by hyperlipidemia, the cerebral blood flow (CBF) was measured. To assess blood–brain barrier integrity, albumin leakage in middle cerebral artery (MCA) area was determined. To evaluate the effect of the drugs on impaired microvessels, the number and morphology of microvessels were assessed in hippocampus area. To further evaluate the ultrastructure of microvessels in hippocampus, transmission electron microscopy (TEM) and scanning electron microscopy (SEM) were carried out. To assess the profiles of claudin-5 and occludin in hippocampus, we performed immunofluorescence. Finally, to assess the expression of claudin-5, JAM-1, occludin and ZO-1 in hippocampus, western blot was carried out. The results showed that YXQNW, SC, and YXQNW + SC improved cognitive impairment of aged LDLR (+/-) golden Syrian hamsters without lowering plasma TC and LDL-C. YXQNW, SC, and YXQNW + SC attenuated albumin leakage in MCA area and neuronal damage in hippocampus, concomitant with an increase in CBF, a decrease of perivascular edema and an up-regulated expression of claudin-5, occludin and ZO-1. In conclusion, YXQNW, SC, and YXQNW + SC are able to improve cognitive ability in aged LDLR (+/-) golden Syrian hamsters via mechanisms involving maintaining blood–brain barrier integrity. These findings provide evidence suggesting YXQNW or SC as a potential regime to counteract the cognitive impairment caused by familial hypercholesterolemia.

## Introduction

Familial hypercholesterolemia is a severe autosomal dominant genetic disease mainly caused by LDLR gene deficiency (Abul-Husn et al., 2016). It is characterized by elevated level of plasma TC and LDL-C (Goldberg et al., 2011). A large part of homozygous FH patients die of myocardial infarction in their teenage years (Kastelein et al., 2007), while heterozygous FH patients suffer from similar diseases in their middle age and at old age (Yuan et al., 2006). Recently, it is reported that aged heterozygous FH patients are more inclined to suffer from dementia compared with control people, which can be attenuated by long-term statin therapy or plasma exchange (Hyttinen et al., 2010; Orehek, 2016). However, long-term statin therapy is reported to have side effects, such as rhabdomyolysis, hepatic injury, and renal failure (Mukhtar and Reckless, 2005; Kromer and Moosmann, 2009; Kareem et al., 2017). Plasma exchange is a very expensive therapeutic strategy for many patients. Thus, it is imperative to develop a novel therapeutic to deal with dementia for FH patients.

The Blood–brain barrier (BBB) is situated between brain tissue and blood. It is composed of cerebral vascular endothelial cells, surrounded by pericytes and astrocytic endfeet (Goldstein, 1988). The TJ proteins between adjacent endothelial cells are very important for its barrier function (Abbott et al., 2010). TJ proteins consist of three types of transmembrane proteins including claudin-5, occludin, and junction adhesion molecules-1 (JAM-1). Several scaffolding proteins like ZO-1 connect to the actin skeleton (Wolburg et al., 2009; Abbott et al., 2010). The transmembrane TJ proteins between adjacent brain microvascular endothelial cells were chimeric. They connected to the cytoskeleton protein β-actin with the help of ZO-1 (Coisne and Engelhardt, 2011; Tietz and Engelhardt, 2015).

Several researchers showed that BBB injury was involved in dementia (Ringelstein and Nabavi, 2005; Montagne et al., 2015; van de Haar et al., 2015). Recent studies have demonstrated that high cholesterol and high low density lipoprotein (LDL) in plasma are two risk factors of BBB injury (Lin et al., 2010; Ehrlich and Humpel, 2012; Schreurs and Cipolla, 2014). The underlying mechanism is related to the down-regulation of TJ proteins (Lin et al., 2010; ElAli et al., 2011). Perivascular edema results in a collapse of capillaries, leading to decrease of cerebral perfusion, neuronal damage, and memory deficiency (Ringelstein and Nabavi, 2005; Huang et al., 2012; Mao et al., 2015). Thus, besides lowering plasma TC and LDL-C, attenuating BBB injury in hippocampus, especially up-regulating the expression of TJ proteins may be another option to attenuate dementia induced by FH or hypercholesterolemia.

YangXue QingNao Wan (YXQNW) is a compound Chinese medicine produced by Tasly Pharmaceutical Co., Ltd. (Tianjin, China). It is approved by the Chinese State Food and Drug Administration (Z20063808) for application in China to deal with neurological disorders, such as dizziness, headache or vertigo. YXQNW and Cerebralcare Granule (CG) are in different formulation, but have the same ingredients, including *Radix angelicae sinensis* (Dang Gui), *Rhizoma chuan xiong* (Chuan Xiong), *Radix paeoniae alba* (Bai Shao), *Ramulus uncariae cum uncis* (Gou Teng), *Caulis spatholobi* (Ji Xue Teng), *Spica prunellae* (Xia Ku Cao), *Concha margaritifera usta* (Zhen Zhu Mu), *Radix rehmanniae preparata* (Di Huang), *Semen cassiae* (Jue Ming Zi), *Rhizoma corydalis yanhusuo* (Yan Hu Suo), and *Herba asari* (Xi Xin) (Huang et al., 2012).

Our previous studies have demonstrated that CG can inhibit the production of reactive oxygen species (ROS), alleviate the microcirculatory disturbances and neuronal damage caused by I/R in Mongolian gerbils (Xu et al., 2009; Sun et al., 2010). Also, CG can attenuate I/R induced brain edema through influencing the TJ protein degradation and caveolin-1 expression in vascular endothelial cells (Huang et al., 2012). A recently published study reported that CG attenuates D-galactose induced memory impairment in mice (Qu et al., 2016). However, no study has been published as to the effect of CG or YXQNW on memory impairment induced by FH or hyperlipidemia.

Silybin is the primary active ingredient in the seed extracts of the *Silybum marianum* (Shui Fei Ji). It is discovered as the first member of a new natural compounds called flavonolignans, and widely used in many countries to treat hepatic disease caused by, for example, snakebites, insect stings, mushroom poisoning, and alcohol, etc. (Abenavoli et al., 2010; Biedermann et al., 2014). SC (Tasly Pharmaceutical Co., Ltd, Tianjin, China) is a silybin-phospholipid complex with silybin as the bioactive component, and currently used in China for the patients with acute or chronic hepatitis and fatty liver disease.

Our previous studies have found that SC can attenuate the non-alcoholic fatty liver disease (NAFLD) induced by high-fat-diet (HFD) through inhibiting *de novo* lipogenesis and promoting fatty acid oxidation in hamsters (Cui et al., 2017). Also, recent studies have demonstrated that silybin improves learning and memory ability in LPS-treated rats and SAMP8 mice (Joshi et al., 2014; Jangra et al., 2015; Jin et al., 2016). But whether SC can improve memory deficiency in hypercholesterolemia animals is still unclear.

LDLR (+/-) golden Syrian hamster is a new animal model for heterozygous FH bred by Prof. Liu (Gao et al., 2014). By using this model, the present study investigated the effect of YXQNW, SC, and YXQNW + SC on cognitive impairment induced by FH, and further gained insight into its underlying mechanism.

## Materials and Methods

### Animals

Female LDLR (+/-) golden Syrian hamsters and their wild type (WT) control hamsters, aged 14 months and weighing 150–200 g, were provided by Cardiovascular Institute of Peking University Health Science Center. The animals were housed at 23 ± 1°C and humidity of 40 ± 5% under a 12-h light/dark cycle. The hamsters were operated on according to the guidelines of the Peking University Health Science Center Animal research committee, and all the experiment procedures were approved by Peking University Biomedical Ethics Committee Experimental Animal Ethics Branch (LA2017214).

### Drugs

YXQNW (batch number 160345) and SC (batch number 16085449, 35 mg silybin in each capsule) were provided by Tasly Pharmaceutical Co., Ltd. (Tianjin, China). They were dissolved in normal saline (NS) to a concentration of 100 mg/ml and 1 mg/ml, respectively, before use.

YXQNW is consisted of multiple components. The effects of the drug depend on the synergy of different components. Thus the pharmaceutical companies usually use tetrahydropalmatine as the representative compound to show the ADME (absorption, distribution, metabolism, and excretion) of YXQNW. The half-life of YXQNW in plasma (t1/2) is 7.22 ± 2.03 h, while the entire recipe’s half-life (t1/2) is 4.45 h.

The plasma concentration of SC has been tested, showing that in the healthy people taking 360 mg silybin lecithin complex (Silibinin), the peak value of blood concentration was 298 + 96 ng/ml with the peak time appearing at 1.6 + 0.3 h, and the average residual time in blood was 3.6 + 0.4 h.

### Experimental Groups

Sixty eight LDLR (+/-) golden Syrian hamsters with 68 WT control hamsters were included and randomly divided into 8 groups, 17 animals each: (1) control + NS group, (2) control + YXQNW group, (3) control + SC group, (4) control + YXQNW + SC group, (5) LDLR (+/-) + NS group, (6) LDLR (+/-) + YXQNW group, (7) LDLR (+/-) + SC group, (8) LDLR (+/-) + YXQNW + SC group. Number per group (17) is the sum of the animal number required for each experiment in that group (please see **Table 1** for details). All the drugs were administrated once daily by gavage for 30 days. The dose of YXQNW was 0.5 g/kg/day, while SC was 0.1 g/kg/day. The animals in NS groups received the equal volume of NS in the same manner.

**Table 1:** Number of animals for different experimental groups and various parameters.

	Control	Control	Control	Control	LDLR (+/-)	LDLR (+/-)	LDLR (+/-)	LDLR (+/-)	Total
	+ NS	+ YXQNW	+ SC	+ YXQNW + SC	+ NS	+ YXQNW	+ SC	+ YXQNW + SC	
Plasma TC, TG, LDL-C, HDL-C	9	9	9	9	9	9	9	9	72
CBF and Albumin leakage	(6)	(6)	(6)	(6)	(6)	(6)	(6)	(6)	(48)
Electron microscopy	(3)	(3)	(3)	(3)	(3)	(3)	(3)	(3)	(24)
Y maze	8	8	8	8	8	8	8	8	64
Nissl stain, immunohistochemistry and immunofluorescence	(4)	(4)	(4)	(4)	(4)	(4)	(4)	(4)	(32)
Western blot	(4)	(4)	(4)	(4)	(4)	(4)	(4)	(4)	(32)
Total	17	17	17	17	17	17	17	17	136

*The animals used for CBF, albumin leakage and electron microscopy were the same as those for detection of plasma TC, TG, LDL-C, HDL-C. The animals used for Nissl staining, immunofluorescence, immunohistochemistry, and western blot were the same as those for detection of Y maze task.*

### Determination of Plasma TC, TG, LDL-C, and HDL-C

The blood was collected before and after administration of drugs via right canthus vein after the hamsters (*n* = 9) were fasted for 12 h. The samples were centrifuged at 2500 *g* for 10 min at 4°C to separate plasma. The concentrations of plasma TC, triglyceride (TG), LDL-C, and HDL-C were assessed following the kit instruction (BioSino Bio-Technology and Science Inc., Beijing, China).

### Y Maze Task

Y maze task was performed based on the method described previously (Kitanaka et al., 2015; Ghafouri et al., 2016) with some modifications (*n* = 8). The Y maze used in this experiment was consisted of three dark gray acrylic arms (40 cm long, 18 cm wide, and 30 cm high) that were intersected at 120°. The hamsters were put into the neutral zone and allowed for moving freely for 8 min. During that time, entries into all arms were noted (four paws had to be inside the arm for a valid entry) and a spontaneous alternation was counted if an animal entered three different arms consecutively. Percentage of spontaneous alternation was calculated according to following formula: [(number of alternations)/(total number of arm entries - 2)] × 100%. The Y maze was wiped clean between trials with 10% ethanol. Y maze task was carried out both before and after treatment.

### Cerebral Blood Flow Measurement

After anesthetized, CBF of animals (*n* = 6) was measured using laser Doppler perfusion image system (PeriScan PIM3; PERIMED, Stockholm, Sweden). In brief, an incision was made through the scalp, and the skin was retracted to expose the skull. The periosteal connective tissue adherent to the skull was removed with a sterile cotton swab. A computer-controlled optical scanner directed a low-powered He-Ne laser beam over the exposed parietal bone. The scanner head was positioned in parallel to the cerebral cortex at a distance of 18.5 cm. At each measuring site, the laser beam could reach the cortex through parietal bone lacking blood vessels.

### Albumin Leakage Assessment

After finishing the CBF measurement, the hamster’s head was secured in a stereotactic frame. With a hand-held drill, a 4 mm × 6 mm cranial window was made through an incision 1 mm behind the coronal suture and 1 mm on the right side of the sagittal suture. This location corresponds to the MCA area. The dura was removed and the pia mater was superfused contiguously with 37°C saline. Then the hamsters were intravenously injected with fluorescein FITC-labeled albumin (Sigma Chemical, St. Louis, MO, United States) at a dose of 50 mg/kg body weight, and the albumin leakage from venule in the MCA area was observed using an upright fluorescence microscope (BX51WI, Olympus, Tokyo, Japan) equipped with a color monitor (20PF5120, Philips, Eindhoven, Netherland), and a DVD recorder (DVR-560H, Philips Eindhoven, Netherland). We magnified the venules in MCA area for 40 and 200 times. The time between preparation of cranial window and observations was 10 min. Only those hamsters whose cranial windows were without any bleeding or inflammatory manifestation were included in the study. The fluorescent intensities within the venules (*I*_v_) and in the perivenular interstitial area (*I*_i_) were measured with ImageJ (Bethesda, MD, United States) software. Albumin leakage was presented as *I*_i_*/I*_v_.

### Tissue Preparation for Histology

Anesthetized hamsters (*n* = 4) were perfused through the left ventricle with 0.9% saline followed by 4% paraformaldehyde (PFA) dissolved by 0.1 M PBS for 40 min. Brains were removed and kept in the same fixative overnight. The samples were infused in 30% sucrose at 4°C for 2 days, and then embedded in Tissue-Tek OCT compound (Miles Inc., Elkhart, IN, United States), frozen in 2-methylbutane which was cooled in liquid-nitrogen. Coronal brain sections were cut with a cryostat microtome (CM1900, Leica, Nussloch, Germany) at -20°C, and thawed and mounted onto gelatin-coated slides. After air-dried, the brain slides were stored at -20°C.

### Nissl Staining

For Nissl staining, the sections were stained with cresyl violet acetate (Sigma-Aldrich, St. Louis, MO, United States) and examined with light microscope (BX512DP70, Olympus, Japan) according to the standard procedure.

### Immunohistochemistry

For immunohistochemistry, the sections were incubated with mouse anti-CD31 (1:50, Thermo Scientific, MA1-80069, Waltham, United States) diluted in PBS overnight at 4°C after blocking with bovine serum albumin. Then the samples were incubated with a biotinylated secondary antibody followed by avidin-biotin-peroxidase complex. Positive staining was visualized with diaminobenzidine. The CA1 region was magnified for 40 times and 200 times, captured by a digital camera connected to a microscope (BX512DP70, Olympus, Tokyo, Japan). And the number of open microvessels was analyzed with Image-Pro Plus 5.0 software (IPP, Media Cybernetic, Bethesda, MD, United States). Five fields of CA1 region were examined for each animal (Tian et al., 2013).

### Immunofluorescence

For immunofluorescence, slices were treated with 0.01 M sodium citrate for antigen retrieval and washed by PBS for three times. After blocking with normal goat serum at room temperature for 0.5 h, the slices were incubated with primary antibodies diluted in PBS overnight at 4°C. The primary antibodies applied were as follows: mouse anti-claudin-5, mouse anti-occludin (1:50, Invitrogen, Camarillo, CA, United States), and rabbit anti-VWF (1:200, Millipore, Temecula, CA, United States). After washed by PBS for three times, the slides were incubated with secondary antibodies diluted in PBS for 2 h at 37°C. The secondary antibodies used were as follows: Dylight 488-labeled goat anti-rabbit IgG (1:100, KPL, Gaithersburg, MD, United States) and Dylight 549-labeled goat anti-mouse IgG (1:100, KPL, Gaithersburg, MD, United States). Hoechst 33342 (Molecular Probes) was applied to stain the nuclei. Finally, the brain sections were mounted, coverslipped, and photographed under a laser scanning confocal microscope (TCS SP5, Leica, Mannheim, Germany).

### Ultrastructure Examination

The brains of hamsters (*n* = 3) were removed after perfusing with 3% glutaraldehyde in 0.1 M PBS at a speed of 3 ml/min for 40 min. Then the hippocampi of the hamsters were dissected. For TEM, the hippocampi were cut into a slice of about 1 mm thick and placed in freshly prepared 3% glutaraldehyde overnight at 4°C. After washing with 0.1 M PBS for three times, the tissues were post-fixed in 1% osmium tetroxide in 0.1 M PBS for 2 h at 4°C. Then the samples were dehydrated and embedded in Epon 812. Ultra-thin sections of hippocampus were stained with uranium acetate and lead citrate and examined in a transmission electron microscope (JEM 1400 plus, JEOL, Tokyo, Japan). For SEM, the samples were cut into blocks and placed in the freshly prepared 3% glutaraldehyde for 2 h, rinsed with 0.1 M PBS for 2 h. The specimens were processed as routine and examined under a scanning electron microscope (JSM-5600LV, JEOL, Tokyo, Japan).

### Western Blot

Western blot analysis (*n* = 4) was performed. Briefly, whole-cell protein was prepared from isolated hippocampus and extracted by RIPA buffer. Protein samples were separated using a 10% Tris-HCl precast gel for polyacrylamide electrophoresis (Bio-Rad Laboratories, Hercules, CA, United States) at 80 V for 150 min. Then the proteins were transferred to PVDF membranes with 220 mA at 4°C for 120 min. The membranes were blocked in TBST containing 3% non-fat milk for 1 h at room temperature and then incubated overnight at 4°C with primary antibodies against claudin-5, occludin (1:1000, Invitrogen, Camarillo, CA, United States), JAM-1 and ZO-1 (1:200, Santa Cruz Biotechnology, Santa Cruz, CA, United States). The membranes were washed with TBST for three times before incubation with the respective horseradish peroxidase-conjugated secondary antibody (1:3000, Santa Cruz Biotechnology, Santa Cruz, CA, United States) at room temperature for 60 min. Then the membranes were washed with TBST for three times again and antibodies binding was detected using enhanced chemiluminescence detection kit (applygene). Band intensities were quantified by densitometry and expressed as mean area density using ImageJ (Bethesda, MD, United States) software.

### Data Analyses

All data were expressed as mean ± SEM. Statistical analysis was conducted by one-way ANOVA or two-way ANOVA, followed by Bonferroni test. A value of *p* < 0.05 was considered statistically significant.

## Results

### YXQNW and SC Have No Effect on Plasma TC, TG, LDL-C, and HDL-C in Aged LDLR (+/-) Hamsters

**FIGURE 1:**
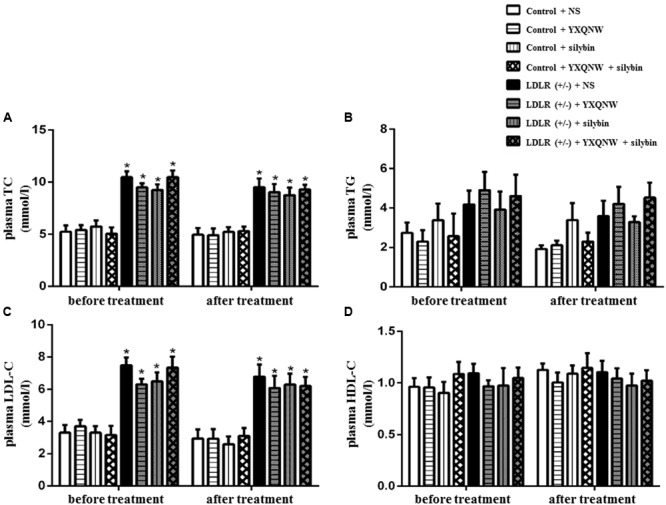
The effect of YXQNW, SC, and YXQNW + SC on plasma lipid in different groups. **(A–D)** Respectively, present the plasma TC, TG, LDL-C, and HDL-C level before and after treatment in all groups. Values are mean ± SEM. ^∗^*p* < 0.05 vs. control + NS group, ^#^*p* < 0.05 vs. LDLR (+/–) + NS group, *n* = 9.

To investigate whether the two drugs have effect on blood lipid level, we measured plasma TC, TG, LDL-C, and HDL-C both before and after administration of drugs. **Figure 1** shows that before treatment, compared with WT animals, the plasma TC and LDL-C were elevated significantly in LDLR (+/-) animals (*p* < 0.05, *n* = 9) (**Figures 1A,C**), while plasma TG and HDL-C were not significantly different (*p* > 0.05, *n* = 9) (**Figures 1B,D**). After administering of the drugs for 30 days, comparing with LDLR (+/-) + NS group, treatment with YXQNW or SC or YXQNW + SC had no significant effect on the level of plasma TC and LDL-C (*p* > 0.05, *n* = 9) (**Figures 1A,C**). These results showed that treatment with YXQNW, SC, or YXQNW + SC could not influence the plasma lipid levels of aged LDLR (+/-) golden Syrian hamsters.

### YXQNW and SC Improve Learning and Memory Ability in Aged LDLR (+/-) Hamsters

**FIGURE 2:**
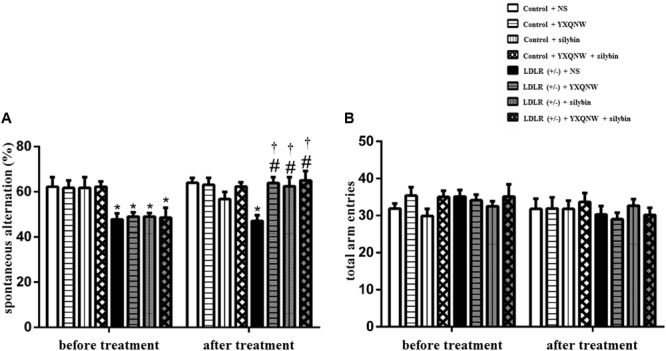
The effect of YXQNW, SC, and YXQNW + SC on spontaneous alternation **(A)** and total arm entries **(B)** in hamsters tested in a Y maze task. Values are mean ± SEM. ^∗^*p* < 0.05 vs. control + NS group, ^#^*p* < 0.05 vs. LDLR (+/–) + NS group, ^†^*p* < 0.05 vs. before treatment, *n* = 8.

To evaluate the effect of the drugs on dementia caused by FH, we next used Y maze task to assess the behavioral indicator of learning and memory before and after treatment. **Figure 2** illustrates the spontaneous alternation and total arm entries assessed by Y maze for hamsters in the eight experimental groups. Every hamster went through Y maze task both before and after treatment. **Figure 2A** shows that before treatment, the spontaneous alternation in the LDLR (+/-) animals was significantly lower than in the WT animals (*p* < 0.05, *n* = 8). After administration for 30 days, spontaneous alternation in LDLR (+/-) + NS group remained at a low level as before treatment, while YXQNW, SC, and YXQNW + SC elevated spontaneous alternation, significantly, to a level close to WT (*p* < 0.05, *n* = 8). **Figure 2B** shows no difference in the total arm entries found among groups both before and after treatment (*p* > 0.05, *n* = 8). These results indicated that YXQNW, SC, and YXQNW + SC had a significant improving effect on learning and memory ability in aged LDLR (+/-) golden Syrian hamsters.

### YXQNW and SC Increase the Cerebral Blood Flow in Aged LDLR (+/-) Hamsters

**FIGURE 3:**
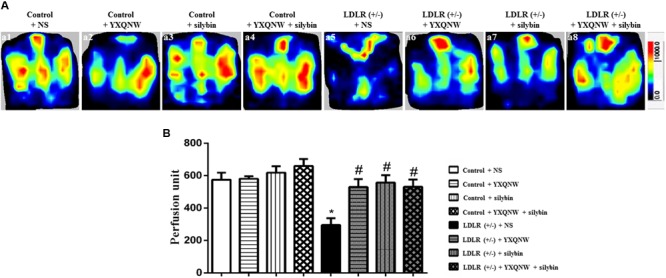
The effect of YXQNW, SC, and YXQNW + SC on CBF. **(A)** The representative laser-Doppler perfusion images of control + NS group **(a1)**, control + YXQNW group **(a2)**, control + SC group **(a3)**, control + YXQNW + SC group **(a4)**, LDLR (+/–) + NS group **(a5)**, LDLR (+/–) + YXQNW group **(a6)**, LDLR (+/–) + SC group **(a7)** and LDLR (+/–) + YXQNW + SC group **(a8)**, respectively. The magnitude of CBF is represented by different colors, with blue to red denoting low to high. **(B)** Quantitative analysis of CBF in different groups, values are mean ± SEM. ^∗^*p* < 0.05 vs. control + NS group, ^#^*p* < 0.05 vs. LDLR (+/–) + NS group, *n* = 6.

Hyperlipidemia may impact blood vessels leading to a disturbed blood flow which contributes to cognition impairment. CBF was thus determined in different groups by a laser Doppler perfusion image system (**Figures 3A,B**). A heat map representation of the CBF in LDLR (+/-) + NS group showed markedly lower perfusion than in control + NS group (**Figure 3A**). Quantification showed a decrease of 50% in CBF compared to control + NS group (*p* < 0.05, *n* = 6) (**Figure 3B**). Treatment with either YXQNW, SC, or YXQNW + SC markedly increased the CBF, close to WT animal values (*p* < 0.05, *n* = 6). These results suggested the potential of these drugs to attenuate the impaired blood vessels in aged LDLR (+/-) hamsters.

### YXQNW and SC Reduce Albumin Leakage in Middle Cerebral Artery Area in Aged LDLR (+/-) Hamsters

**FIGURE 4:**
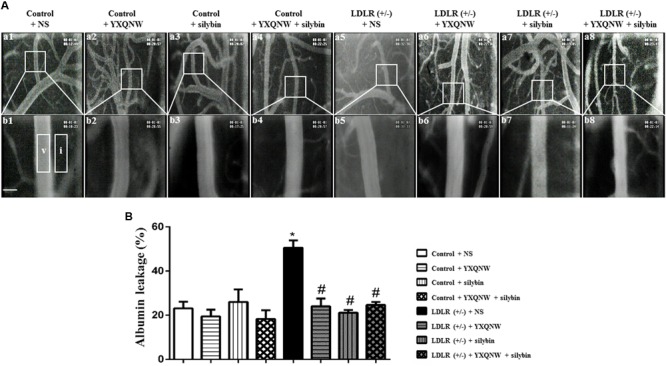
YXQNW, SC, and YXQNW + SC reduce albumin leakage from cerebral venules. **(A)** Representative images of albumin leakage from venules in control + NS group **(a1,b1)**, control + YXQNW group **(a2,b2)**, control + SC group **(a3,b3)**, control + YXQNW + SC group **(a4,b4)**, LDLR (+/–) + NS group **(a5,b5)**, LDLR (+/–) + YXQNW group **(a6,b6)**, LDLR (+/–) + SC group **(a7,b7)**, and LDLR (+/–) + YXQNW + SC group **(a8,b8)**, respectively, wherein **(b)** is the high magnification of the area inside the boxes in **(a)**. Rectangles represent the areas for determination of fluorescence. V, cerebral venule. I, interstitial tissue. Bar = 50 μm. **(B)** Statistic analysis of albumin leakage in different groups. Values are the mean ± SEM. ^∗^*p* < 0.05 vs. control + NS group, ^#^*p* < 0.05 vs. LDLR (+/–) + NS group, *n* = 6.

Hyperlipidemia-impaired cerebral vessels may manifest BBB breakdown. We thus evaluated the albumin leakage from venules in MCA area. **Figure 4A** shows the representative pictures of *trans*-vascular flux of FITC-labeled albumin from cerebral venules in all groups. The quantitation of the albumin leakage in each group is depicted in **Figure 4B**, demonstrating that compared with control + NS group, the FITC-labeled albumin leakage from cerebral venules significantly increased in LDLR (+/-) + NS group, which was attenuated remarkably by treatment with YXQNW, SC, or YXQNW + SC (*p* < 0.05, *n* = 6). This result confirmed the potential of the two drugs to alleviate BBB breakdown in aged LDLR (+/-) hamsters.

### YXQNW and SC Increase the Number of Open Microvessels in the Hippocampus of Aged LDLR (+/-) Hamsters

**FIGURE 5:**
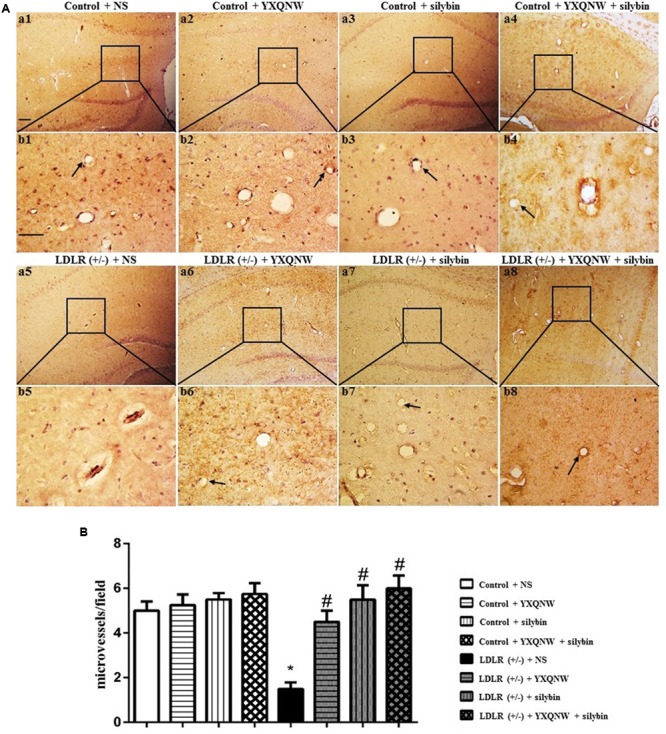
The effect of YXQNW, SC, and YXQNW + SC on the number of open microvessels in hamster hippocampus. **(A)** Representative immunohistochemistry images at the hamster hippocampus of control + NS group **(a1,b1)**, control + YXQNW group **(a2,b2)**, control + SC group **(a3,b3)**, control + YXQNW + SC group **(a4,b4)**, LDLR (+/–) + NS group **(a5,b5)**, LDLR (+/–) + YXQNW group **(a6,b6)**, LDLR (+/–) + SC group **(a7,b7)**, and LDLR (+/–) + YXQNW + SC group **(a8,b8)**, respectively, wherein **(b)** is the high magnification of **(a)**. Bar = 100 μm. Arrows indicate open calliparies. **(B)** Quantitative evaluation of CD31-positive open microvessels. Values are the mean ± SEM. ^∗^*p* < 0.05 vs. control + NS group, ^#^*p* < 0.05 vs. LDLR (+/–) + NS group, *n* = 4.

The effect of the drugs on impaired microvessels in aged LDLR (+/-) golden Syrian hamsters was further assessed by morphologic methods. For this, an immunohistochemical staining for CD31 was performed to delineate the vessels in hippocampus, a region more relevant to cognition. **Figure 5A** shows the representative images between CA1 and dentate gyrus region stained by CD31 in all the groups, with the statistic result for the number of opening microvessels (arrows) in each group presenting in **Figure 5B**. Apparently, compared to control group (**Figures 5Aa1,b1**), LDLR (+/-) + NS group (**Figures 5Aa5,b5**) exhibited a significantly reduced number of open microvessels with numerous collapsed microvessels, accompanying with obvious peri-vascular edema. YXQNW, SC, or YXQNW + SC treatment minimized the perivascular edema and protected the reduction in the number of the open microvessels dramatically (**Figures 5Aa6,b6,a7,b7,a8,b8**). The quantitative evaluation of the number of open microvessels confirmed the survey results (*p* < 0.05, *n* = 4). These results offered morphological evidence for the ability of the drugs to protect microvessels from collapsing in aged LDLR (+/-) golden Syrian hamsters.

### YXQNW and SC Ameliorate the Cerebral Microvasculature in Hippocampus of Aged LDLR (+/-) Hamsters

**FIGURE 6:**
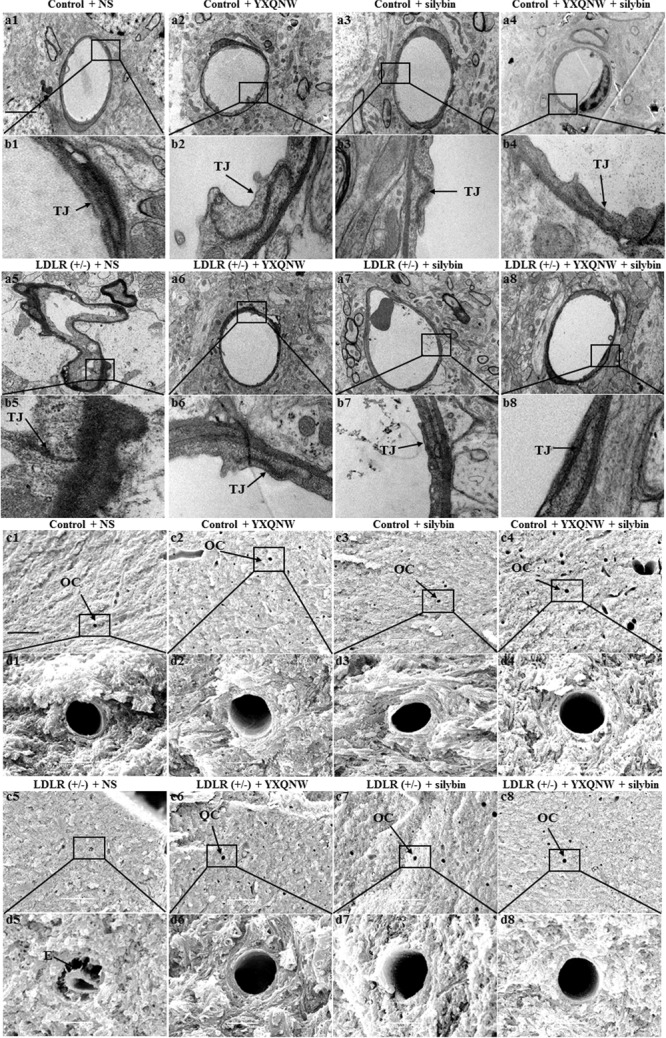
Ultrastructure of microvessels in the hippocampus of hamsters in different groups. The representative transmission electron micrographs of capillaries in the hippocampus are shown in **(a,b)**, while the scanning electron micrographs of the hippocampus shown in **(c,d)**. The micrographs in **(b,d)** are high magnification of the area inside the boxes in **(a,c)**, respectively, with 1 denoting control + NS group, 2 control + YXQNW group, 3 control + SC group, 4 control + YXQNW + SC group, 5 LDLR (+/–) + NS group, 6 LDLR (+/–) + YXQNW group, 7 LDLR (+/–) + SC group and 8 LDLR (+/–) + YXQNW + SC group, Bar = 50 μm in **(a,c)**. TJ, tight junction. E, edema. OC, open capillaries.

The beneficial role of the two drugs on impaired vessels in aged LDLR (+/-) golden Syrian hamsters was supported by electron microscopy. **Figures 6a,b** show the representative images of TEM in the hippocampus of hamsters from different groups. Compared to control + NS group (**Figures 6a1,b1**), the microvasculature in LDLR (+/-) + NS group revealed a remarkable alteration, such as rough inner surface, separation of the basal membrane, and appearance of edema around the vessels (**Figures 6a5,b5**). YXQNW, SC, and YXQNW + SC medication attenuated these changes (**Figures 6a6,b6,a7,b7,a8,b8**). **Figures 6c,d** show the representative images of SEM in the hippocampus of hamsters in all the groups. Microvessels in WT animals showed a normal morphology, while in LDLR (+/-) + NS group we observed a rough inner surface of microvessels and some perivascular cavities. This change was attenuated by YXQNW, SC, and YXQNW + SC administration. This result further verified our observations obtained by TEM and supported the protective effect of the two drugs on impaired cerebral vessels in aged LDLR (+/-) hamsters.

### YXQNW and SC Protect Against the Neuronal Damage in CA1, CA2, CA3, and DG Region of Hippocampus in Aged LDLR (+/-) Hamsters

**FIGURE 7:**
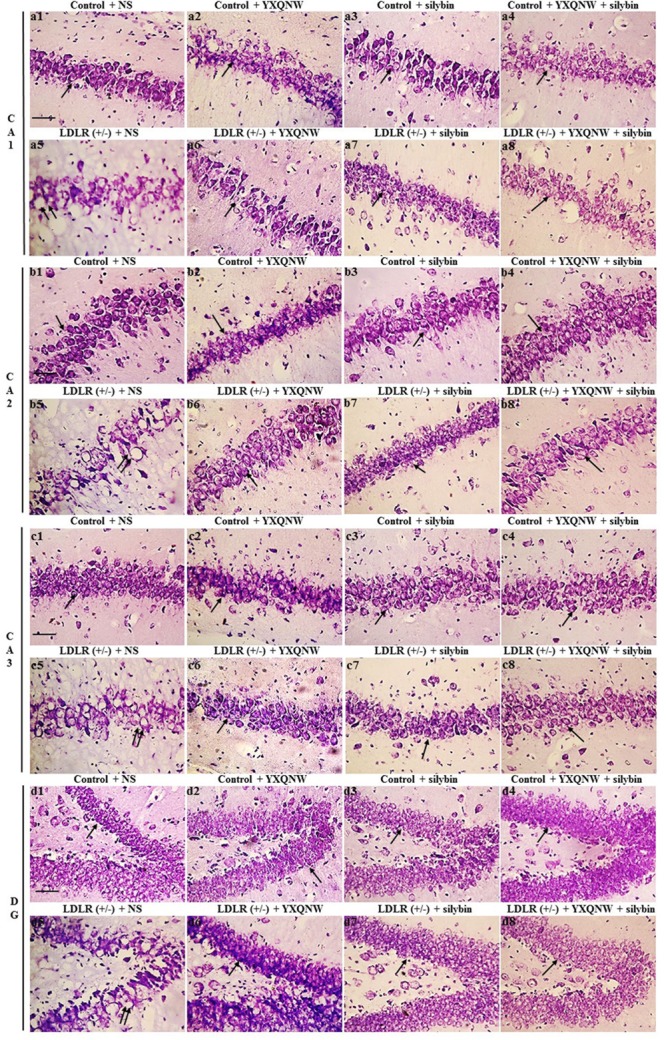
The effect of YXQNW, SC, and YXQNW + SC on the number of neurons in hamster hippocampus. Tissue sections are stained with cresyl violet for Nissl stain. **(a–d)** Respectively, present the representative images of the CA1, CA2, CA3, and DG area in different groups, with 1 denoting control + NS group, 2 control + YXQNW group, 3 control + SC group, 4 control + YXQNW + SC group, 5 LDLR (+/–) + NS group, 6 LDLR (+/–) + YXQNW group, 7 LDLR (+/–) + SC group and 8 LDLR (+/–) + YXQNW + SC group. Bar = 50 μm. The arrows indicate Nissl-positive neurons, and the double arrows indicate nuclear pyknosis with karyorrhexis.

We assessed the effect of the two drugs on hippocampal neuronal damage in aged LDLR (+/-) hamsters and thus its ability to attenuate cognition impairment. **Figure 7** shows the representative images of Nissl staining in all groups. In CA1, CA2, CA3, and DG region of hippocampus, neurons of control + NS group (**Figures 7a1,b1,c1,d1**) showed normal morphological features, while those in LDLR (+/-) + NS group presented diverse neuronal damages such as cell swelling, nuclear pyknosis and karyorrhexis (**Figures 7a5,b5,c5,d5**). YXQNW (**Figures 7a6,b6,c6,d6**), SC (**Figures 7a7,b7,c7,d7**) and YXQNW + SC (**Figures 7a8,b8,c8,d8**) treatment effectively prevented the hippocampal neuronal damages in aged LDLR (+/-) hamsters. This result suggested the potential of the two drugs to alleviate neuronal damage in the hippocampus of aged LDLR (+/-) golden Syrian hamsters thus attenuate cognition impairment.

### YXQNW and SC Alleviate Degradation of Tight Junction Proteins Claudin-5, Occludin, ZO-1 in the Hippocampus of Aged LDLR (+/-) Hamsters

**FIGURE 8:**
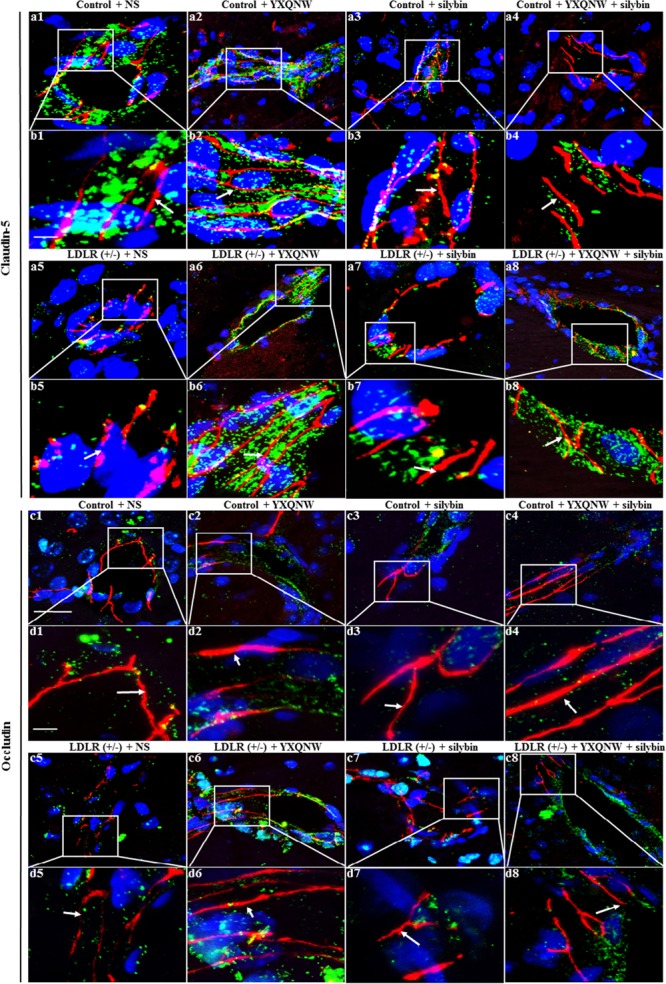
The effect of YXQNW, SC, and YXQNW + SC on the expression and distribution of tight junction proteins of microvessels in hamster hippocampus. Shown are the representative immunofluorescence confocal images of claudin-5 **(a1–a8,b1–b8)** and occludin **(c1–c8,d1–d8)**. Claudin-5 (red) and occludin (red) localized at the junction of endothelial cells with marker VWF (green). Blue color denotes nuclei. The area within the rectangle in each picture **(a,c)** is enlarged and presented below **(b,d)** correspondingly. The number 1 denoting control + NS group, 2 control + YXQNW group, 3 control + SC group, 4 control + YXQNW + SC group, 5 LDLR (+/–) + NS group, 6 LDLR (+/–) + YXQNW group, 7 LDLR (+/–) + SC group and 8 LDLR (+/–) + YXQNW + SC group. White arrows indicate the localization of claudin-5 and occludin. Bars = 25 μm in **(a,c)**, Bars = 50 μm in **(b,d)**.

**FIGURE 9:**
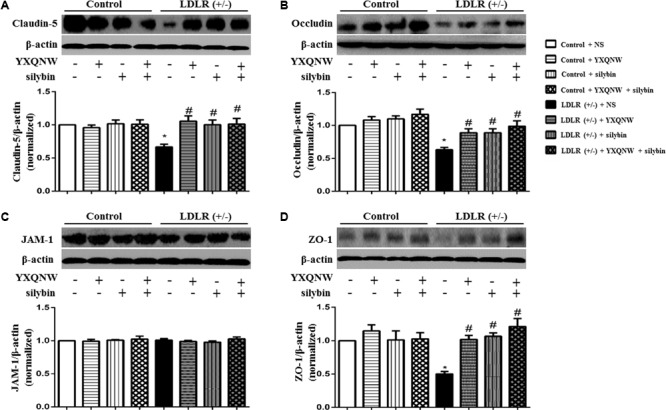
The effect of YXQNW, SC and YXQNW + SC on the expression of tight junction proteins in the hamsters hippocampus. **(A)** Representative western blot bands and quantitative assessment of claudin-5. **(B)** Representative western blot bands and quantitative assessment of occludin. **(C)** Representative western blot bands and quantitative assessment of JAM-1. **(D)** Representative western blot bands and quantitative assessment of ZO-1. All the quantifications were undertaken based on the data of three independent experiments and normalized to β-actin, respectively. Values are the mean ± SEM. ^∗^*p* < 0.05 vs. control + NS group, ^#^*p* < 0.05 vs. LDLR (+/–) + NS group, *n* = 4.

To further identify the role of YXQNW and SC in maintaining BBB integrity in hippocampus, confocal microscopy and western blot were used to examine the vascular endothelial TJ proteins. Confocal microscopy revealed that both claudin-5 and occludin localized between endothelial cells as continuous lines in control + NS group (**Figures 8a1,b1,c1,d1**), control + YXQNW group (**Figures 8a2,b2,c2,d2**), control + SC group (**Figures 8a3,b3,c3,d3**) and control + YXQNW + SC group (**Figures 8a4,b4,c4,d4**). While in LDLR (+/-) + NS group (**Figures 8a5,b5,c5,d5**), these continuous distributions were disrupted apparently, becoming dotted lines, concomitant with reduction in immune staining, indicating degradation of the TJ proteins claudin-5 and occludin in aged LDLR (+/-) hamster. Interestingly, this degradation was alleviated by YXQNW (**Figures 8a6,b6,c6,d6**), SC (**Figures 8a7,b7,c7,d7**), and YXQNW + SC (**Figures 8a8,b8,c8,d8**) treatment. These results were confirmed by western blot (*p* < 0.05, *n* = 4) (**Figures 9A,B**). We further examined the expression of JAM-1 and ZO-1, the other two important components of TJ proteins in hippocampus by western blot, and observed that the expression pattern of ZO-1 (**Figure 9D**) was similar to that of claudin-5 and occludin (*p* < 0.05, *n* = 4), while the expression of JAM-1 in hippocampus tissue did not change significantly among the eight experiment groups (*p* > 0.05, *n* = 4) (**Figure 9C**). These results indicated that YXQNW, SC, and YXQNW + SC could alleviate the reduction in TJ proteins expression of aged LDLR (+/-) golden Syrian hamsters, which may account for the protective role of the drugs in BBB breakdown.

## Discussion

An appropriate animal model is critical to research disease pathogenesis and drug testing, particularly for a valuable extrapolation of the outcomes to clinical practice. LDLR (+/-) golden Syrian hamster is a newly established animal model for FH. Hamsters have many similarities to humans in lipid metabolism. For example, they have a high level of plasma CETP, a low level of hepatic low-density lipoprotein receptor (LDLR) activity and a high glycemic response to dietary fructose. All of which are not observed in other rodents such as mice and rats (Briand, 2010; Naples et al., 2012; Gao et al., 2014). Using this model, the present study demonstrated that both YXQNW and SC are capable of attenuating the cognitive impairment of the aged LDLR (+/-) hamsters. To gain insight into the rationale behind their beneficial role, we first examined the effect of the two medicines on plasma lipid and revealed that SC had no effect on the level of TC and LDL-C in aged LDLR (+/-) hamsters. This result is in contrast to a previously published study showing that silibinin attenuated hyperlipidaemiaun in rats (Gobalakrishnan et al., 2016). This difference is most likely due to the different animal models used. We believe that the result of the present study is more feasible to translate to humans because the lipid metabolism of the model is more similar to humans.

As a medication mainly used to deal with brain ailments, YXQNW has not been reported to exert any effect on hyperlipidaemia. In line with this, no effect of YXQNW on the hypercholesterolemia was observed in LDLR (+/-) golden Syrian hamsters alike. Of interest, despite the negative finding with respect to the role of YXQNW and SC in attenuating hypercholesterolemia, both drugs were found to alleviate the cognitive impairment of LDLR (+/-) golden Syrian hamsters, implying the occurrence of a mechanism other than lowering plasma cholesterol that mediates the effect of the two drugs on cognitive impairment in the current circumstance. Several researches have reported that hypercholesterolemia is a highly risk factor for BBB injury through down-regulating the expression of TJ proteins (Kalayci et al., 2009; ElAli et al., 2011; Ehrlich and Humpel, 2012). Elevated LDL being oxidated to ox-LDL in plasma may impair BBB integrity in humans as well as in rodents (Kanata et al., 2006; Chen et al., 2007; Ishino et al., 2007). In view of the importance of BBB integrity in maintaining brain homeostasis, we investigated effect of the two drugs on BBB.

Blood–brain barrier consists of a layer of specialized endothelial cells along with basal membrane, surrounded by pericytes and astrocytic endfeet, which prevents circulating toxic components from entering the CNS and allows those essential for neuronal cells to pass through. BBB disruption and alteration in transport and endothelial cell surface proteins may lead to BBB pathology. Several researches have shown that BBB disruption is related to many neurodegenerative diseases. For example, vascular pathologies and BBB disruption has been shown in postmortem samples from AD patients (van de Haar et al., 2015). Of interest, the present study showed that YXQNW and SC, the two medicines each containing distinct ingredients, were able to protect BBB disruption in LDLR (+/-) golden Syrian hamsters with equal efficiency at the dose used, as indicated by the prevention of albumin leakage from cerebral microvasculatures, and the maintaining of the TJ proteins. More importantly, the two medicines exhibited equal benefit as well on the impaired cognitive ability of LDLR (+/-) golden Syrian hamsters, highlighting the BBB disruption as the cause of the cognitive impairment in the present case. This speculation can also explain the fact that no additive effect of combination of the two medicines was observed on the protection of BBB and cognitive ability, a finding that looks a little bit strange at first glance, since administration of any one of them was enough to completely preserve the BBB integrity and thereby to protect cognitive function.

Blood–brain barrier disruption is known to exert detrimental effect on neurons in multiple aspect (Montagne et al., 2015; van de Haar et al., 2015). BBB breakdown leads to accumulation of neurotoxic proteins in central nerve system, including fibrin, thrombin, hemoglobin, iron-containing hemosiderin, free iron, and/or plasmin. BBB disruption results in cerebral edema, which imposes pressure on microvasculatures, leading to reduced blood supply thereby causing energy metabolism deficit. These insults collectively cause progressive neurodegeneration with loss of neurons. Consistent with these, we observed a decreased CBF in LDLR (+/-) golden Syrian hamsters, as well as a reduced number of neurons in the hippocampus, which certainly contributes to cognitive impairment. Of notice, YXQNW, SC or the combination of the two improved the CBF and protected the neurons in hippocampus from loss with equal efficiency, as they did on the preservation of BBB and cognitive ability. which further support the protection of BBB as the mechanism that underlies the beneficial role of the two medicines in cognition protection.

## Conclusion

Our present study showed that both YXQNW and SC have the ability to improve cognitive abilities in aged LDLR (+/-) golden Syrian hamsters. The mechanisms implicated include a protection from BBB breakdown through upregulation of the TJs. This result suggests YXQNW or SC as potential strategy to protect against the cognitive impairment for patients with hyperlipidaemia. Since cognitive impairment was only evaluated using one test in the present study, tests in more comprehensive manner are required to verify this conclusion.

## Author Contributions

Y-YG performed the research, analyzed the data, and wrote the manuscript. PH, Y-YL, and Q-FC contributed to the animal experiments. GL and Y-HW provided the animals and contributed to the measure plasma TC, TG, LDL-C, and HDL-C. MY contributed to the Y maze task. LiY, X-HW, and LeY contributed to the Nissl stain, immunohistochemistry, and immunofluorescence. B-HH, X-RZ, and XC contributed to the electron microscopy. C-SP and Y-CC contributed to the western blotting. C-SW and KS contributed to the other experiments. J-YF and QL revised the manuscript. Z-ZM and J-YH designed and funded the research, interpreted the data, and finally approved the submission of this manuscript. All authors read and agreed with the manuscript.

## Conflict of Interest Statement

The authors declare that the research was conducted in the absence of any commercial or financial relationships that could be construed as a potential conflict of interest.

## Abbreviations

BBB: blood–brain-barrier
CBF: cerebral blood flow
CETP: cholesteryl ester transport protein
FH: familial hypercholesterolemia
HDL-C: high density lipoprotein cholesterol
I/R: ischemia-reperfusion
JAM-1: junctional adhesion molecule-1
LDL-C: low density lipoprotein cholesterol
LDLR: low density lipoprotein receptor
MCA: middle cerebral artery
PBS: phosphate buffered solution
PVDF: polyvinylidene fluoride
SC: silibinin capsules
SEM: scanning electron microscopy
TBST: Tris-buffered saline Tween
TC: total cholesterol
TEM: transmission electron microscopy
TG: total triglyceride
TJ: tight junction
YXQNW: YangXue QingNao Wan
ZO-1: zonula occludens-1
